# Impact of Accidental High or Low Implantation Depth on Peri-Procedural Outcomes after Implantation with the Self-Expanding ACURATE neo2

**DOI:** 10.3390/jcm13175342

**Published:** 2024-09-09

**Authors:** Clemens Eckel, Won-Keun Kim, Judith Schlüter, Matthias Renker, Sophie Bargon, Christina Grothusen, Albrecht Elsässer, Guido Dohmen, Yeong-Hoon Choi, Efstratios I. Charitos, Christian W. Hamm, Samuel Sossalla, Helge Möllmann, Johannes Blumenstein

**Affiliations:** 1 Department of Cardiology, St. Johannes Hospital, 44137 Dortmund, Germany; clemens.eckel@joho-dortmund.de (C.E.);; 2 School VI—School of Medicine and Health Sciences, Carl von Ossietzky Universität Oldenburg, 26129 Oldenburg, Germany; 3 Department of Cardiology, Kerckhoff Heart Center, 61231 Bad Nauheim, Germany; 4 Department of Cardiology, Justus-Liebig University of Giessen, 35390 Giessen, Germany; 5 Department of Cardiac Surgery, Kerckhoff Heart Center, 61231 Bad Nauheim, Germany; 6 Department of Cardiac Surgery, University of Kiel, 24118 Kiel, Germany; 7 Department of Cardiac Surgery, St. Johannes Hospital, 44137 Dortmund, Germany

**Keywords:** TAVR, self-expanding, THV, transfemoral, implantation depth, PPM, PVL, PPI, embolization

## Abstract

**Background**: Precise implantation could play a crucial role in the technical success of transcatheter aortic valve replacement (TAVR) for some prostheses. The impact of an accidental implantation depth (ID) outside the recommended range has not been assessed for the ACURATE *neo2* (NEO2). **Methods**: Data from 1839 patients with severe native aortic stenosis treated with the NEO2 prosthesis were evaluated. We compared the results of prostheses implanted in an ID both inside and outside the recommendations. The outcome assessment followed the Valve Academic Research Consortium-3 criteria. **Results**: Patients were retrospectively divided into high (<3 mm; *n* = 412), optimal (3–7 mm; *n* = 1236), and low (>7 mm; *n* = 169) implantations. Technical success (94.7% vs. 94.7% vs. 91.7%, *p* = 0.296) and device success were high (90.1% vs. 89.3% vs. 84.6%, *p* = 0.112) without differences between groups. Rates of relevant paravalvular regurgitation (PVL; >mild or VinV due to PVL) were comparable (1.2% vs. 1.8% vs. 1.2%, *p* = 0.759). Even when hemodynamics were superior in the high-implantation group, with greater iEOA (1.01 cm^2^/m^2^ vs. 0.95 cm^2^/m^2^ vs. 0.92 cm^2^/m^2^, *p* < 0.001), spontaneous embolization or after post-dilatation was more common. Low implantation was associated with a higher rate of associated pacemaker implantation (PPI) (6.1% vs. 8.8% vs. 14.8%, *p* = 0.001). **Conclusions**: Implantation with the ACURATE *neo2* showed excellent hemodynamic results, including low gradients and a small number of relevant PVL, in line with a high technical success rate that was irrespective of the ID. A favorable outcome can also be achieved in accidental low or high positions. Low implantation was associated with a higher rate of associated pacemaker implantation. Deliberately high implantation should be avoided due to the risk of embolization.

## 1. Introduction

Transcatheter aortic valve replacement (TAVR) represents the gold standard in the treatment of elderly patients with high-grade aortic valve stenosis (AS). There has been steady improvement in transcatheter heart valves (THVs) over the years, but precise implantation of the prosthesis could be a key factor in the technical success of the procedure. In recent years, clinical research has focused on the implementation of standardized implantation concepts such as the cusp-overlap technique to achieve optimal prosthesis implantation depth (ID) [1]. The influence of ID on the conduction system and the rate of permanent pacemaker implantation (PPI) for commercially available prostheses have been well examined [2,3,4]. In contrast, data on prosthesis-specific effects on hemodynamic parameters, such as the occurrence of paravalvular leakage (PVL) and prosthesis-patient mismatch (PPM), are scarce [5]. Each prosthesis has a specific landing zone, as defined in the corresponding instructions for use (IFU). The optimal position in the annulus is usually analyzed in in vitro benchmark tests. However, ID depends on many factors and can be difficult to control in practice. Accidentally high or low implantation of prostheses impacts hemodynamic results, pacemaker rates, coronary access, and embolization rates [6,7,8]. Adverse events, as well as relevant outcome variables, were mentioned according to the Valve Academic Research Consortium-3 criteria [9]. Therefore, we aimed to analyze to what extent the self-expanding (SE) ACURATE *neo2* (NEO2) tolerates an accidental ID outside the recommended landing in a real-world population and what impact it has on hemodynamics and clinical performance.

## 2. Methods

### 2.1. Patient Cohort

A total of 1839 patients undergoing transfemoral TAVR with the NEO2 valve (Boston Scientific, Marlborough, MA, USA) between December 2016 and July 2023 at two German high-volume TAVR centers were included retrospectively. Patients with primary bailout valve-in-valve (VinV) procedures were excluded (*n* = 22). Comorbidities, risk scores, and data on echocardiography, multidetector computed tomography (MDCT), and cardiac catheterization were recorded as baseline characteristics in a dedicated database, as were procedural data and complications. Supplemental follow-up data were collected from outpatient visits, from recent physician reports, or by telephone interview. The study was conducted in accordance with the Declaration of Helsinki. Due to the retrospective nature of this study and the anonymous data processing, ethical approval was waived by each local ethics committee. Patients approved anonymous data collection.

### 2.2. Multidetector Computed Tomography

Multidetector computed tomography (MDCT) was performed with a 64-slice or a 192-slice dual-source scanner (Somatom Definition or Somatom Force, Siemens Healthcare, Forchheim, Germany), as previously described [10]. Analysis of MDCT datasets was accomplished by using dedicated software (3mensio Structural Heart version 10.6, Pie Medical, The Netherlands). The aortic valve calcium score (AVCS) was measured according to the Agatston method using non-contrast-enhanced MDCT scans [11]. Relevant left ventricular outflow tract (LVOT) calcification or the presence of eccentric aortic valve calcification was determined by a visual assessment of the aortic valve in short-axis views. The cover index is defined as: 100 × [(THV diameter − perimeter derived annulus diameter)/THV diameter].

### 2.3. Device Description

The technical features of the NEO2 have already been described [12]. Compared with its predecessor (ACURATE *neo*), the prosthesis has a larger external sealing skirt to mitigate PVL.

### 2.4. Definition and Measurement of Implantation Depth

**Figure 1:**
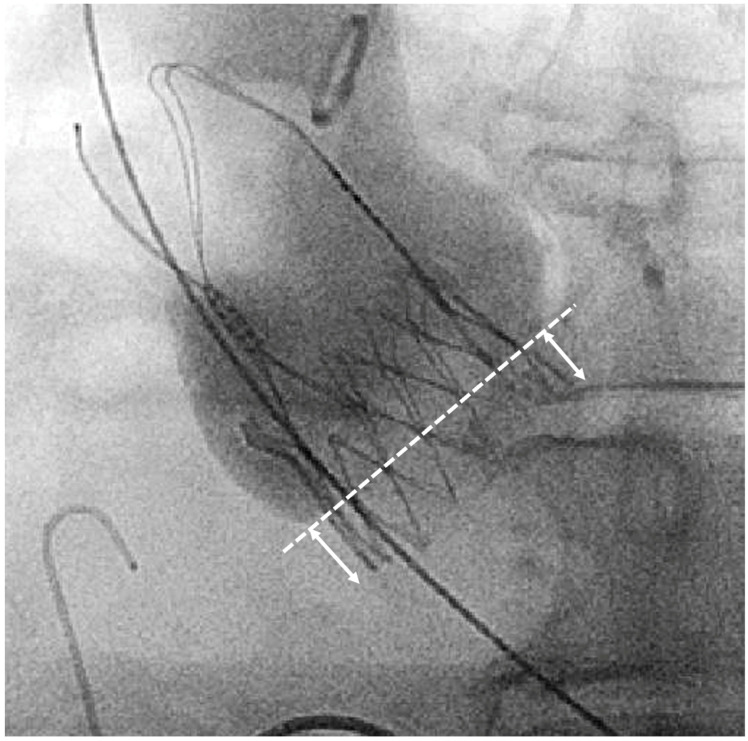
Fluoroscopic measurement of implantation depth at non-coronary-cusp (NCC) in the cusp-overlap view. Arrows indicate implantation depth (ID).

**Figure 2:**
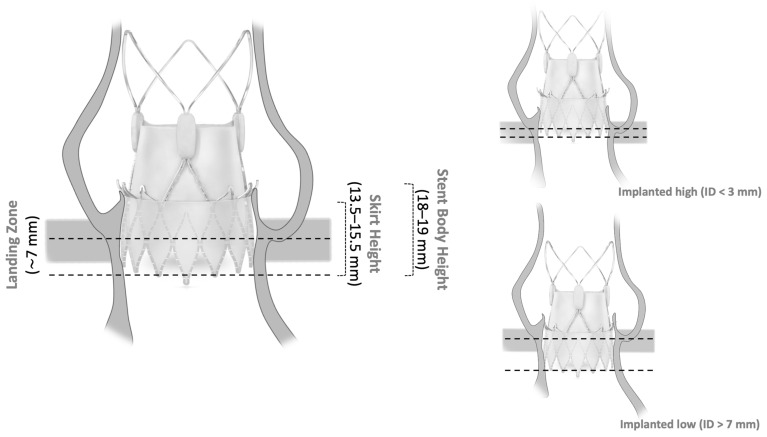
Schematic showing implant depth (ID) according to aortic annulus. Illustration of the ACURATE *neo2* in the native aortic annulus in relation to the ID. The center of each image (**left**; **upper right**; **lower right**) of the prosthesis is copyrighted by the manufacturer (© 2023 Boston Scientific Corporation (Marlborough, MA, USA) or its affiliates. All rights reserved).

The manufacturer recommends a landing zone corridor for SE NEO2. All prostheses were initially intended to be implanted in the recommended landing zone. Assessment of the ID was performed retrospectively in the cusp-overlap view (Figure 1). We rated the implantation based on the measured ID < 3 mm as high, between 3 and 7 mm as recommended, and >7 mm as low (Figure 2).

### 2.5. Outcomes

The primary outcome measures were technical success, device success (30 days), and hemodynamic performance (PVL, PPM, effective orifice area [EOA], and mean gradients) according to the Valve Academic Research Consortium 3 (VARC3) criteria [9]. Secondary outcome measures were procedural duration and contrast amount, as well as in-hospital events, according to VARC 3. PVL was assessed by the respective echocardiography laboratory and graded according to VARC3 as none/trace, mild, moderate, or severe.

### 2.6. Statistical Analysis

Statistical analyses were conducted using R (version 4.2.1, 2021; R Foundation for Statistical Computing, Vienna, Austria). Continuous data are provided as mean (SD) or median (IQR), and categorical variables as frequencies, as appropriate. Comparison of multiple groups was accomplished using the Tukey–Kramer post hoc test for normally distributed variables or otherwise with Benjamini–Hochberg correction, as indicated. The *p*-value for trend was calculated with a Pearson-test for normally distributed variables or, otherwise, with a Spearman-test for non-normally distributed variables. The Mantel–Haenszel test was used for categorical variables. Additional univariate and multivariable logistic regression analyses were performed to evaluate the impact of ID on specific clinical or echocardiographic events (PVL, PPM, and PPI). Covariates with a *p*-value < 0.01 in univariate regression were included in the multivariate model. For all analyses, a two-sided *p*-value < 0.05 was considered significant.

## 3. Results

### 3.1. Baseline Data

A total of 1817 patients (with implantation high, *n* = 412; optimal, *n* = 1236; low, *n* = 169) were included. The mean age was 81.7 [SD ± 5.9] years, 61.1% were female, and the median EuroSCORE II was 3.1 [IQR 2.1–4.9]. Table 1 shows baseline characteristics after selection for implantation depth (high; optimal; low).

### 3.2. Procedural Data and In-Hospital Outcome

There was no significant trend for higher procedural duration (42 min vs. 41 min vs. 45 min, *p* = 0.756) in between groups (Table 2). The rate of post-dilatation was lower in the low implantation group (41.5% vs. 27.9% vs. 24.4%, *p* < 0001). Oversizing was less frequent in the low implantation group (5.9% vs. 4.8% vs. 4.5%, *p* < 0.001). Hemodynamic performance was inferior in patients with low implantation, including smaller indexed EOA (1.01 cm^2^/m^2^ vs. 0.95 cm^2^/m^2^ vs. 0.92 cm^2^/m^2^, *p* < 0.001), and there was a higher rate of severe PPM (2.2% vs. 3.7% vs. 6.4%, *p* = 0.024). Rates of relevant PVL (1.2% vs. 1.8% vs. 1.2%, *p* = 0.759) were comparable between the groups. Technical success (94.7% vs. 94.7% vs. 91.7%, *p* = 0.296) and device success (90.1% vs. 89.3% vs. 84.6%, *p* = 112) rates were also comparable between the groups. The rate of PPI was higher with low implantation (6.1% vs. 8.8% vs. 14.8%, *p* = 0.001). Further results regarding outcomes and complications are provided in Table 2.

Multivariable logistic regression analysis (Table 3, Table 4 and Table 5) revealed an independent association between ID and PPI (OR 1.15 [1.06,1.24], *p* < 0.001). There was no independent association of ID with PPM (OR 1.09 [0.97,1.22], *p* = 0.161) or relevant PVL (OR 1.06 [0.91,1.25], *p* = 0.439).

A device embolization happened in a total of 22 cases, 17 (77.3%) spontaneously or after post-dilatation, and 5 (22.7%) due to mechanical complications. Among cases with spontaneous or post-dilatation embolization, 10 (71.4%) had a primary ID too high or too low, see Supplementary Table S1. In two cases, the primary ID is missing.

## 4. Discussion

The impact of an ID outside the recommended landing zone on outcome and hemodynamics after implantation with the SE NEO2 prosthetic valve is unclear, as data on this topic are scarce. Therefore, we investigated to what extent the NEO2 tolerates IDs outside of the recommended landing zone and what effect the ID has on hemodynamics and outcome.

The main findings of the present study are: (1) NEO2 shows excellent hemodynamic results, including low gradients and even low rates of relevant PVL, which is in line with the high technical success rate; this was found to be irrespective of ID; (2) Accidental high implantation of the prosthesis showed slightly improved hemodynamics in terms of EOA and severe PPM and reduced the need for PPI; (3) Accidental low implantation resulted in a higher rate of severe PPM and PPI; (4) The risk of valve embolization was high in too low or high implantations.

### 4.1. Procedural Performance

The SE supraannular ACURATE *neo2* THV has emerged as a prosthesis with excellent hemodynamic properties and a low rate of PPI [12,13]. Nevertheless, the precise implantation of the device itself also plays a prominent role regarding procedural outcome and hemodynamics. The SE NEO2 is implanted using a top-down technique. After precise positioning at the target level, the stabilization arches are released first, followed by the stent body itself. Despite precise positioning of the marker at the target level, factors like calcification of the native annulus as well as aortal kinking or aortic angle, which can both lead to an unpredictable force on the delivery catheter, might influence the final ID [14,15]. In this context, recent studies have analyzed the effects of rapid pacing during implantation on more precise and optimized ID, which might be an option in difficult anatomies [1,15]. Embolization occurred in a total of 22 cases, in 17 cases spontaneously or after post-dilatation, and in 5 cases due to mechanical complications such as wire problems or during device detachment. Among cases with spontaneous embolization and known primary ID, the implantation was too high (*n* = 6) or too low (*n* = 4) in 71.4% (*n* = 10/14) of the cases, see Supplemental Table S1. The higher rate of post-dilatation in high-implanted valves in our study might be explained by the higher rate of primary paravalvular leakage due to impaired sealing in the native annulus or to secure the valve position to avoid embolization.

### 4.2. Hemodynamic Outcome

While some studies reveal a positive correlation between ID and the rate of PVL with early-generation SE THVs, we were not able to confirm this with the NEO2 [16]. This may be due to the general decrease in PVL after adapting the prosthesis design and adding a skirt in third-generation prostheses [12,13,17]. The most recent comparisons between different SE THVs no longer show a correlation between ID and the occurrence of PVL and confirm our results showing generally low rates of relevant PVL (1.2–1.8%) [18]. An analysis of the hemodynamics of the Neo family in small annuli revealed an increased risk of PPM for implantation at low ID [19]. Event rates in high- or low-implantation may be influenced by differences in the baseline characteristics, such as, for example, eGFR, LVEF, mean gradient, or EuroScore II. However, multivariable regression analyses fail to show an independent association of ID with moderate or severe PPM, whereas data on balloon-expandable prostheses show a positive correlation between ID and the risk of PPM [20]. The fact that EOA decreases with increasing ID is in contrast to the lack of an effect of ID on post-interventional gradients. This might be related to the general problem of echocardiographic assessment of post-interventional hemodynamics [21]. Analyses of the impact of PVL and PPM on long-term survival currently have not produced consistent results [22]. However, the Scope I trial found no difference regarding mortality over the long term, despite an increased rate of PVL with the predecessor, the ACURATE *neo* [23].

### 4.3. Permanent Pacemaker Implantation

Generally, low implantation, short membranous septum (MS), preexisting right bundle branch block (RBBB), and AV block I are known predictors of the necessity for PPI [3,5,18]. A patient-specific ID according to the length of the MS (MIDAS; Minimizing Depth According to the membranous Septum) has been shown to reduce the rate of PPI with the Medtronic Evolut THV system [24]. In addition, the cusp-overlap technique has proven to be valuable, as this view has shown a high ID most accurately at the level of the MS for the Medtronic Evolut THV, although these results have not been confirmed for the ACURATE *neo* [1,25]. Overall, the NEO 2 is well known for its low PPI rates due to the special stent design and the low radial force [26]. In line with this, our results show a range of PPI rates between 6.1% and 14.8%, depending on ID. The right bundle branch block as well as the ID are proven to impact the PPI rate in multivariable analysis, irrespective of differences in the baseline characteristics.

### 4.4. Coronary Access

High implantation with supraannular, long-stent frame SE devices impairs future coronary access [6,27,28]. In our study, periinterventional coronary obstruction occurred in only one patient (0.6%). The topic of future coronary access with respect to the ID of the NEO 2 systems has to be evaluated further, especially in the context of future VinV options. More recently, a modified implantation strategy that focuses on the correct anatomical rotation has been applied in practice. This has further reduced the risk of acute coronary obstruction and facilitated access to future coronary interventions [29,30].

### 4.5. Limitations

The present study should be viewed in light of some limitations: its retrospective, non-randomized nature; the absence of external monitoring of adverse events; the lack of core laboratory analysis of imaging data such as the echocardiographic assessment of PVL; the visual assessment of LVOT calcification and eccentric aortic valve calcification without further quantification; and the fact that the ID was measured angiographically and not by computed tomography.

## 5. Conclusions

The self-expanding ACURATE *neo2* shows excellent hemodynamic results, including low gradients and low rates of relevant PVL in line with high technical success rates. Interestingly, these favorable results were also consistent for final positions outside the recommended landing zone, including accidental low and high positions. Deliberately high implantation should be avoided due to the risk of device embolization.

## Figures and Tables

**Table 1:** Baseline characteristics according to implantation position.

Variable	High (ID < 3 mm)	Optimal (ID 3–7 mm)	Low (ID > 7 mm)	*p* Value
	*n* = 412	*n* = 1236	*n* = 169	
Age, years	82.0 [78.0; 85.0]	82.0 [79.0; 86.0]	82.0 [79.0; 85.0]	0.352
Female sex, %	225 (54.6%)	770 (62.3%)	116 (68.6%)	0.001
BMI, kg/m^2^	26.2 [23.8; 30.0]	26.2 [23.6; 29.8]	26.6 [24.2; 30.1]	0.877
EuroSCORE II, %	2.7 [1.9; 4.6]	3.1 [2.1; 4.8]	3.9 [2.5; 5.8]	<0.001
eGFR, mL/min/1.73 m^2^	62.0 [46.0; 79.0]	60.0 [44.0; 77.0]	53.0 [39.0; 69.0]	<0.001
Peripheral artery disease	47 (11.4%)	138 (11.2%)	27 (16.0%)	0.267
Prior stroke	54 (13.1%)	149 (12.1%)	23 (13.6%)	0.920
Atrial fibrillation	151 (36.7%)	513 (41.5%)	77 (45.6%)	0.029
Coronary artery disease	227 (55.1%)	792 (64.1%)	112 (66.3%)	0.001
Prior coronary intervention	138 (33.6%)	461 (37.3%)	69 (40.8%)	0.076
Echocardiographic data				
Left ventricular ejection fraction, %	65.0 [59.0; 65.0]	64.0 [55.0; 65.0]	60.0 [50.0; 65.0]	<0.001
Mean aortic valve gradient, mmHg	44.0 [36.0; 53.0]	41.0 [31.0; 48.0]	37.0 [29.0; 46.0]	<0.001
EOA, cm^2^	0.7 [0.6; 0.8]	0.7 [0.6; 0.9]	0.8 [0.6; 0.9]	0.057
Electrocardiographic data				
Right bundle branch block	44 (10.7%)	97 (7.9%)	17 (10.2%)	0.411
Left bundle branch block	48 (11.7%)	85 (6.9%)	14 (8.4%)	0.029
Atrioventricular block	78 (18.9%)	204 (16.6%)	34 (20.5%)	0.916
MDCT data				
Annulus diameter, mm	23.8 [22.8; 25.0]	23.9 [22.7; 25.1]	24.0 [22.7; 25.3]	0.600
LVOT, mm	22.6 [21.1; 24.1]	23.2 [21.4; 24.8]	23.2 [22.1; 25.1]	<0.001
STJ, mm	28.5 [27.0; 30.2]	27.7 [26.0; 29.8]	27.5 [25.6; 29.7]	<0.001
Aortic valve calcification, AU	2723 [1930; 3756]	2110 [1417; 3031]	1803 [1176; 2793]	<0.001
Calcification in LVOT	47 (11.4%)	119 (9.6%)	19 (11.2%)	0.636
Eccentric calcification	50 (12.2%)	117 (9.5%)	13 (7.7%)	0.061

Abbreviations: BMI = body mass index; eGFR = estimated glomerular filtration rate; EOA = estimated (aortic valve) orifice area; LVOT = left ventricular outflow tract; STJ = sinotubular junction.

**Table 2:** Procedural outcomes and complications according to implantation position.

Variable	High (ID < 3 mm)	Optimal (ID 3–7 mm)	Low (ID > 7 mm)	*p* Value
	*n* = 412	*n* = 1236	*n* = 169	
Procedural parameter				
Prosthesis size				0.142
S	92 (22.3%)	336 (27.2%)	50 (29.6%)	
M	173 (42.0%)	483 (39.1%)	60 (35.5%)	
L	147 (35.7%)	417 (33.7%)	59 (34.9%)	
Procedural duration, min	42 [35; 5]	41 [34; 50]	45 [35; 5]	0.756
Contrast agent, mL	38 [20; 70]	58 [24; 100]	96 [56; 120]	<0.001
Pre dilatation, %	387 (93.9%)	1146 (92.8%)	157 (92.9%)	0.517
Post dilatation, %	170 (41.5%)	339 (27.9%)	40 (24.4%)	<0.001
Cover index (annulus), %	5.87 [3.9; 7.4]	4.83 [3.0; 7.0]	4.52 [2.7; 6.7]	<0.001
Echocardiographic outcome				
Left ventricular ejection fraction, %	65 [62; 65]	65 [57; 65]	62 [52; 65]	<0.001
Mean aortic valve gradient, mmHg	8.0 [6.0; 10.0]	8.00 [6.0; 11.0]	8.00 [6.0; 12.0]	0.072
EOA, cm^2^	1.90 [1.6; 2.1]	1.75 [1.5; 2.0]	1.67 [1.5; 2.0]	<0.001
iEOA, cm^2^/m^2^	1.01 [0.9; 1.2]	0.95 [0.8; 1.1]	0.92 [0.8; 1.1]	<0.001
More-than-mild PPM	8 (2.2%)	40 (3.7%)	9 (6.5%)	0.024
Relevant PVL (>mild/trace or VinV)	5 (1.2%)	22 (1.8%)	2 (1.2%)	0.759
Clinical outcome				
Technical success	390 (94.7%)	1170 (94.7%)	155 (91.7%)	0.296
Device success at 30 days	371 (90.1%)	1104 (89.3%)	143 (84.6%)	0.112
30-day mortality	10 (2.7%)	25 (2.2%)	8 (5.1%)	0.335
Conversion to sternotomy	0 (0.0%)	8 (0.7%)	1 (0.6%)	0.180
Multiple valves (VinV)	0 (0.0%)	2 (0.2%)	0 (0.0%)	0.731
Major vascular complication	14 (3.4%)	32 (2.6%)	1 (0.6%)	0.069
Bleeding (type 3–4)	15 (3.7%)	69 (5.6%)	17 (10.1%)	0.004
Major cardiac structural complication	3 (0.7%)	10 (0.8%)	2 (1.2%)	0.635
Overt CNS injury	8 (2.0%)	38 (3.1%)	5 (3.0%)	0.329
AKI (type 2–4)	6 (1.5%)	23 (1.9%)	5 (3.0%)	0.268
New permanent pacemaker ^1^	23 (6.1%)	96 (8.8%)	23 (14.8%)	0.001

Abbreviation: EOA = estimated (aortic valve) orifice area; iEOA = indexed estimated orifice area; PVL = paravalvular leak; CNS = central nervous system; VinV = Valve-in-Valve; PPM = prosthesis-patient mismatch; AKI = acute kidney injury. ^1^ Excluded patients with pacemakers at baseline.

**Table 3:** Multivariable regression analysis (PPI).

	Univariate	*p* Value	Multivariable	*p* Value
Predictors for pacemaker implantation (PPI)				
Age	1.01 (0.71, 1.43)	*p* = 0.959		
Sex (male), %	0.99 (0.97, 1.02)	*p* = 0.654		
Depth at non-coronary cusp, mm	1.13 (1.04, 1.22)	*p* = 0.002	1.15 (1.06, 1.24)	*p* < 0.001
Aortic valve calcification, AU	1.00 (1.00, 1.00)	*p* = 0.284		
LVOT calcification, %	1.38 (0.83, 2.3)	*p* = 0.228		
Post dilatation, %	0.92 (0.63, 1.35)	*p* = 0.670		
Right bundle branch block, %	6.74 (4.51, 10.07)	*p* < 0.001	7.15 (4.76, 10.75)	*p* < 0.001

**Table 4:** Multivariable Regression Analysis (PVL).

	Univariate	*p* Value	Multivariable	*p* Value
Predictors for relevant paravalvular regurgitation (PVL)				
Age	1.05 (0.98, 1.12)	*p* = 0.182		
Sex (male), %	0.82 (0.38, 1.77)	*p* = 0.611		
Depth at non-coronary cusp, mm	1.05 (0.9, 1.22)	*p* = 0.557	1.06 (0.91, 1.25)	*p* = 0.439
Aortic valve calcification, AU	1.00 (1.00, 1.00)	*p* = 0.181		
LVOT calcification, %	1.41 (0.49, 4.10)	*p* = 0.545		
Eccentric AV calcification, %	2.95 (1.24, 7.00)	*p* = 0.026	3.07 (1.28, 7.33)	*p* = 0.012
Post dilatation, %	1.41 (0.66, 3.01)	*p* = 0.379		
Cover index, %	0.99 (0.99, 1.01)	*p* = 0.790		

**Table 5:** Multivariable Regression Analysis (PPM).

	Univariate	*p* Value	Multivariable	*p* Value
Predictors for severe prosthesis patient mismatch (PPM)				
Age	1.05 (0.98, 1.12)	*p* = 0.182		
Sex (male), %	0.65 (0.36, 1.16)	*p* = 0.133		
Depth at non-coronary cusp, mm	1.11 (0.98, 1.24)	*p* = 0.084	1.09 (0.97, 1.22)	*p* = 0.161
Aortic valve calcification, AU	0.99 (0.99, 1.00)	*p* = 0.135		
LVOT calcification, %	1.41 (0.49, 4.10)	*p* = 0.545		
Post dilatation, %	0.37 (0.17, 0.79)	*p* = 0.004	0.41 (0.19, 0.88)	*p* = 0.022
Cover index, %	1.00 (0.97, 1.04)	*p* = 0.820		
Annulus perimeter, mm	0.87 (0.74, 1.02)	*p* = 0.075	0.88 (0.76, 1.04)	*p* = 0.139

## Data Availability

Data is contained within the article.

## Supplementary Materials

The following supporting information can be downloaded at: https://www.mdpi.com/article/10.3390/jcm13175342/s1, Table S1: Patients with primary device embolization.

## Abbreviations

AS	Aortic stenosis
AVCS	aortic valve calcification score
BE	balloon-expandable
ID	implantation depth
iEOA	indexed effective orifice area
LVOT	left ventricular outflow tract
MDCT	multidetector computed tomography
MS	membranous septum
PPM	prosthesis-patient mismatch
PPI	permanent pacemaker implantation
PVL	paravalvular leakage
SE	self-expanding
TAVR	transcatheter aortic valve replacement
VinV	valve-in-valve
VARC	Valve Academic Research Consortium
