# Supplementary material for: Impact of Accidental High or Low Implantation Depth on Peri-Procedural Outcomes after Implantation with the Self-Expanding ACURATE neo2
Source: J Clin Med. 2024 Sep 9;13(17):5342. doi: 10.3390/jcm13175342 (PMC11396697; doi:10.3390/jcm13175342)
Supplement: Supplementary file 1 [file jcm-13-05342-s001.zip › jcm-3144607-supplementary.pdf]

Supplemental Table S1: Patients with primary device embolization

Patient	avcs	bailout	initial ID	direction	mechanism
**Spontaneous embolization (or after post dilatation)**					
2395	5035	2nd Valve	normal	aorta	post dilatation
944	996	2nd Valve	high	aorta	spontaneous
1721	4368	VinV	low	ventricular	spontaneous
1946	5166	AKE	high	aorta	post dilatation
1976	10033	VinV	high	aorta	spontaneous
2070	4274	VinV	low	ventricular	spontaneous
4899	1690	AKE	*na*	ventricular	spontaneous
5059	2436	AKE	normal	ventricular	spontaneous
5076	1481	VinV	normal	aorta	post dilatation
5181	NA	VinV	high	aorta	post dilatation
5337	1568	VinV	*na*	aorta	spontaneous
2891	5077	VinV	low	ventricular	spontaneous
5376	2687	SIV	high	aorta	spontaneous
3034	1414	AKE	low	ventricular	spontaneous
5707	1969	AKE	*na*	ventricular	spontaneous
5830	3953	none	high	aorta	spontaneous
**Device or wire associated embolization**					
4046	770	2nd Valve	normal	aorta	device attached
4091	2226	SIV	normal	aorta	device attached

Patient	avcs	bailout	initial ID	direction	mechanism
2224	2349	VinV	high	aorta	device attached
2409	2226	2nd Valve	high	aorta	wire associated
2428	486	AKE	normal	ventricular	while VinV due to AI

Abbreviation: avcs = aortic valve calcification; ID = implantation depth; VinV = Valve-in-Valve; AKE = aortic valve replacement; SIV = Stent-in-Valve.
